# Tubercular Peritonitis, with Report of Cases Apparently Cured by Laparatomy1Read at the thirty-first annual meeting of the Medical Association of Centra New York, at Auburn, October 18, 1898.

**Published:** 1899-05

**Authors:** Thomas Jameson

**Affiliations:** Rochester, N. Y.


### TUBERCULAR PERITONITIS, WITH REPORT OF CASES APPARENTLY CURED BY LAPARATOMY.[1]

BY THOMAS JAMESON, M. D.,
Rochester, N. Y.

OUR text-books, when describing a disease generally begin with the definition, the etiology, the morbid anatomy, the symptomatology, diagnosis, the prognosis and treatment with, perhaps, describing a case occasionally by way of illustration.

1. Read at the thirty-first annual meeting of the Medical Association of Centra New York, at Auburn, October 18, 1898.

In choosing my subject for this paper, I wish merely to call your attention to the importance of tubercular peritonitis and to the relative frequency with which it occurs in young women. To do this, I shall reverse the ordinary method of dealing with the description of a disease and describe an actual case first and merely incidently refer to the general subject.

CASE I.—Two years ago I was called to see a young girl in a neighboring village who had been suffering for a year from a slowly progressing enlargement of the abdomen. I found a young woman, aged 19, a pure blond with that bright eye and scarlet flush on the cheeks which we so often associate with tuberculosis. She gave me the following history :

She had always been in good health until twelve months previously, when she noticed her abdomen increasing in size; she consulted a physician who treated her for intestinal indigestion; her condition steadily grew worse; her menses became more and more scanty until they ceased entirely; she had no nausea or vomiting; she had slight, indefinite pains in the abdomen, fair appetite, bowels regular, temperature normal. For some weeks previously she had lost flesh and developed a slight cough. Examination of the lungs revealed fluid in the right pleural cavity. The abdomen was so distended, apparently from fluid, that it was impossible to determine whether there was any localised collection of fluid or not. The patient was removed to the hospital where I aspirated about a quart of fluid from her right pleural cavity. Opening the abdomen a day or two later a large quantity of clear serum escaped. The whole peritoneum was studied with tubercles, in fact it gave a sensation of great roughness to the examining hand. The ovaries and the fimbriated extremities of both tubes were apparently very much disorganised, in fact so much so that a surgeon who was present advised and urged their removal, but nothing was removed. The cavity was thoroughly flushed with a hot saline solution and a glass drainage-tube inserted in the posterior cul-de-sac. The patient's temperature went up after the operation to 103 degrees, but in a day or two it dropped to normal. The tube was removed in forty-eight hours and the patient had an uneventful recovery and rapidly regained her strength, taking nineteen raw eggs a day during her last week at the hospital.

Three months ago, just two years after her operation, the patient presented herself at my office, being apparently in the best of health and feeling quite strong. Her menses were regular and natural and her cough had disappeared. The only thing she suffered from was a hernia in the scar where the drainage had been inserted. Wishing to have that repaired, she once more submitted to an operation and so I was enabled to look into the peritoneum and to notice the condition. It was apparently normal and not a trace of tubercle was to be seen anywhere nor where there any adhesions, but I found a cyst in the right broad ligament, which I removed, but which did not show any evidence macroscopically at least of tubercular affection.

The patient made a good recovery from her second laparatomy and is at present at her home in robust health.

This case I looked upon as worth reporting, because there are but few cases on record where the apparent cures of peritoneal tuberculosis were verified by actual inspection.

By a curious coincidence I had two other cases of the same trouble within a few months.

Case II.—A girl of 22 years, who also had a cough with profound anemia and great loss of strength. Having the previous case in mind I examined her very carefully and found a small amount of fluid in the pleural cavity, but could find no further evidence of lung trouble. On palpating the abdomen there was evidence of fluid present. On operation, the same state of affairs was found as in the previous case, but the patient's condition became so poor under the anesthetic that the pelvic organs were not thoroughly examined. Mere drainage was followed by the same happy results. The patient is today earning her living as a domestic and able to do a hard day's work.

My third case was almost identical with the first two, except that in this case the patient complained of a great deal of abdominal pain and I was able to get a faint history of tuberculosis, her sister having died of pulmonary consumption a few months previously. This patient is also alive and in fair, although not robust health.

In regard to the diagnosis of this condition, it has been pointed out by Henoch and others that it is quite possible to find tubercles in the peritoneum, which are identical in appearance with the tubercles produced by Koch's bacilli and yet are merely little granules of fibrous tissue, resulting from a simple nontubercular peritonitis and in order to positively assert that any given case is really one of tubercular peritonitis it would be necessary to find Koch's bacilli in the tubercles, or else to prove their specific origin by inoculation experiment, but I still maintain that the above cases were truly tubercular, although no microscopical examination was made, for we had clinical symptoms present which were strong corroborative evidence—namely, the presence of fluid in the pleural cavity, the absence of peritoneal pain, the absence of any history of an acute attack of peritonitis, which could result in a simple chronic condition. It also may be stated that some pathologists claim that these simple fibrous granules are really tubercular in their origin, but have undergone fibrous change as a part of a spontaneous cure.

In a case of exploratory laparatomy performed by Halstead, of Baltimore, tubercular peritonitis was found and the cavity was washed out with a normal salt solution and drained. The patient made a good recovery. Several months later, this patient having died of pneumonia, an examination showed the tubercles present containing bacilli, but undergoing a fibroid change

It may be very naturally asked why should a mere laparatomy have a curative influence in such a grave condition ?

There is quite a literature on the subject and a great many theories have been advanced, none of which are satisfactory. In 1895, Jordan connected together fourteen cases in which opening the abdomen has been recorded as including either an anatomical healing or distinct improvement in peritoneal tuberculosis.

Results of inoculation experiments on dogs show that laparatomy will stop peritoneal tuberculosis, if not too far advanced, and also shows that whereas in those cases which have resulted fatally there was not a sign of adhesive inflammation, adhesions were present in all those that had had an operation and arrest of the tubercular process therefrom, whether partial or complete, was accompanied by inflammation.

Prof. Adami, in answer to my inquiries on the subject, writes that his view is this : That what happens after laparatomy corresponds very closely to what is seen in the action of cantharidin and even of tuberculin—namely, the mere fact of operation develops an increased inflammatory action.

Now such inflammatory action is not, as is too frequently thought by surgeons, an evidence of an injurious process; rather it must be regarded as an attempt at repair, whether the attempt is successful or not is another matter, but the process tends to be beneficial to the economy and a slight, simple inflammation set up in an affected abdominal cavity may be sufficient to turn the scale, and to cause the various processes already passing around the tubercles, the pouring out of leucocytes, of serum and so on, to be now sufficiently powerful to bring about destruction of the tubercle bacilli and thereby it is that the actual laparatomy is frequently found beneficial.

If we, for example, observe what happens in lupus when cantharidin or tuberculin is given, the most marked change is to be seen in the exaltation of the inflammatory action and it is by that process that healing is to be brought about.

DISCUSSION.

Dr. Van De Warker, Syracuse, N. Y.: I wish to say something that cannot be brought too forcibly to the attention. I criticise, however, the title of the doctor's paper somewhat. In fact, it uses the word "peritonitis." I think the word "tuberculosis" covers the situation; at least, it is a debatable question whether peritonitis is a primary product or whether it is a secondary result.

The second idea is about the word "cure" I think that is entirely problematical, and it is questionable whether we ought to hold out such an inducement to a patient to undergo operation for tuberculosis as a full and permanent cure. It is very true that some of the patients are cured in a certain sense. I remember of operating in 1887, and that patient is still alive today, but I have operated before and the tubercular process went on and the patient died. My own theory about the curative effect of laparatomy is in view of asepsis, in the first place. I have never yet opened the abdomen where it was broadly distended with fluid without finding large quantities of sere-lymph diffused through the abdomen. Washing out in large quantities, I think, is a very essential feature of the operation. I do not use sterilised saline solution, but simply boiled water, and get the same good results.

In the report of post mortems—the post mortems made with a general view in the dead house of Bellevue hospital—by Dr. Loomis, he there found a large percentage of cases of evident cure of being tuberculosis, showing that the persons had been afflicted at some time with tuberculosis of the lungs. That cure consisted in a pigmentation of the lungs and the fibrous encysting of the tubercles or the granulations, and that occurs, I think, in the peritoneum after operation. That was demonstrated in a case reported by Osler, in which a girl had some months previous to her death, which occurred from pneumonia, been operated on for tuberculosis of the peritoneum. There the same pigmentation of the peritoneum was found and the same fibrous granular encysting of the tubercles. The rest afforded by the relief from pressure and the thorough evacuation of the lymph, which was diffused through the peritoneum, in my mind, explained the curative results.

Dr. Jacobson, Syracuse, N. Y.: One year ago this matter came up before our society, I believe, when we were at Buffalo, and various views were expressed at that time, but the opinion prevailed on all sides that in some unknown way an incision into the abdominal cavity led to the cure of many of these cases.

It is very evident from what the two preceding physicians have said that their methods differ widely. The writer of the paper feels that drainage is essential—the commentator that flushing is necessary. It has never been my practice to either drain or flush, and yet I can recall few cases in which there was present a serous fluid in the abdominal cavity and in which, coincidently, the intestines, the mesentery and other abdominal structures were studded with tubercles, presenting all of the macroscopical appearances of tubercular disease, which did not do well.

My earliest positive case dates back about ten years. This young woman still enjoys good health, although I have thought it wise to put her upon cod liver oil each winter.

Men of the widest experience and highest authority seem agreed that the essential thing to do is to open the abdominal cavity and do as little else as possible.

The only additional step I take is to mop out the cavity. This is done deliberately, yet no time is wasted in doing it, and then the wound is closed.

I do not think we can explain why or how the recovery is brought about, but the clinical fact that simply opening the abdominal cavity and removing the accumulated fluid leads to a cure has been demonstrated over and over again.

The association of a pleural with an abdominal effusion is often seen. Dr. Elsner and myself have been interested in the question in Syracuse, as to whether the pleural effusion would disappear after incision into the abdominal cavity and removal of the ascitic accumulation, and, indeed, we have found this frequently to occur.

We must not overlook the fact that, as the underlying cause of the peritoneal effusion is a tubercular one, there may be tubercular disease in other parts of the body, coexisting or even responsible for it. One of the most interesting cases of which I know was not operated upon, but the ascitic fluid was withdrawn by trocar. At the autopsy there was revealed, in addition to the peritoneal tuberculosis, tubercular disease of the bladder, as well as double tubercular pyonephrosis and tubercular pericarditis, while the primary site of disease was a spinal caries, which had infected some retro-peritoneal glands, and in turn aroused the further manifestations mentioned.

The suppurating forms of tubercular peritonitis are of much more serious nature. I recently had occasion to operate upon a young man from this city, who was sent to us, and in whom we found a large abscess, with cheesy pus, placed between coils of intestines. While, therefore, I should say that as a rule the cases with serous effusions recover, these forms of abdominal tuberculosis with purulent collections rarely do.

If by the term "cure" we mean the permanent removal of the diseased condition, I hardly feel that we are ready yet to say that this is brought about; but that cases apparently get well, and perhaps never have a return of the trouble of sufficient severity as to cause them any serious annoyance, is a fact.

Dr. Creveling, Auburn, N. Y.: I wish to say that I do not believe we know very much about how that cure takes place. We see cases recorded of cures due merely to injections of some sort of fluid, as saline or sterilised water. We also see cases recorded cured from injections of sterilised air. And these reports are made by good, straight men, too.

Then, Mr. President, by merely opening the abdomen and exposing the bowels, the contents of the abdomen, to the air undoubtedly does, in many instances, effect a cure. I think Dr. Morris, of New York, has demonstrated that more thoroughly than any other man in the country, probably any other living man. But you take these severe cases where the bowels are all agglutinated together, where everything is adherent, and I am thoroughly satisfied that merely opening the abdomen does not cure them.

Less than two years ago I performed an operation where the whole abdominal contents were agglutinated in one general mass. As far as possible I tore up all the adhesions between the coils of the intestines. Down about the cecum they were so firm, I could not destroy them. Since that time I have had the privilege of making a post mortem on that fellow. He died from general tuberculosis. Wherever I had torn up the adhesions the bowels were perfectly normal, all the inflammatory process had stopped, the bowels appeared in a perfectly normal condition. Where I had not, the inflammatory condition still existed and the deposit of tubercles, of course, was present. To me it was an instructive case. And I believe, as I said a year ago, in all instances where that can be done it is the proper thing to do, to liberate all the adhering bowel; that is, to break up the adhesions between all the intestines and give them a good, liberal exposure. I do not believe that the shock in the operation is greatly enhanced by such a procedure either. I do not believe it is any greater by performing the operation as quickly as possible, than it is to expose the bowels to the air as long as many operators do, to receive a continued atmospheric influence on the abdominal contents.

Dr. Charles E. Congdon, Buffalo, N. Y.: I wish to compliment the doctor upon his success with his cases.

My experience has led me to believe that the disease recurs in the majority of cases, or that the patients succumb sooner or later in the cure, brought about by intestinal obstruction, due to adhesions. When the disease attacks the intestines, it is usually in the same locality that is invaded in typhoid. That variety which comes under the surgeon's notice forms ulcers, which are usually confluent or circular.

I recall a case that came under my notice a few months ago. As soon as I opened the abdomen the small bowel presented with an ulcer completely encircling it; as soon as it was carefully brought outside it separated to the mesentery. The tubercular process had destroyed everything except a few fibers of the muscular coat, which formed a sort of mesh-work. I resected and did an end-to-end anastomosis. The patient made an uninterrupted recovery.

In another case—I do not know whether one can be pardoned for it or not—I opened the abdomen, expecting to have a cystic tumor to deal with. This patient was seen by several experienced men, who pronounced it an ovarian cyst, prior to my seeing the case. On opening the peritoneal cavity, it was found, as soon as the fluid was evacuated, that the intestines were studded with miliary deposits, also agglutinated in such a manner as to prevent the fluid from being free in the peritoneal cavity; the thickened broad ligaments, uterus displaced to one side by adhesions, the globular shape of the tumor in the abdomen and the tympanitic note above and at both sides, its apparent mobility and the history of the case that led others besides myself to a faulty diagnosis. This patient died some two or three months subsequent to the operation from intestinal occlusion.

---

The use of liquid air as a cautery is already spoken of favorably. It having a temperature of 312° below zero, its action is, to all intents and purposes, the same as the most powerful actual cautery. It does not really burn, but utterly kills the tissues, leaving a blister not unlike a burn. Hence it has been suggested for cauterisation in surgical practice. It is not only a good deal cheaper than the ordinary cautery, but it is much more efficient; and its action can be absolutely controlled. Indeed, a well-known surgeon has already performed a difficult operation on a cancer case with liquid air, and he has reported the case as cured.—*Tri-State Medical Journal.*

